# Bias against parents in science hits women harder

**DOI:** 10.1057/s41599-023-01722-x

**Published:** 2023-05-04

**Authors:** Fernanda Staniscuaski, Arthur V. Machado, Rossana C. Soletti, Fernanda Reichert, Eugenia Zandonà, Pamela B. Mello-Carpes, Camila Infanger, Zelia M. C. Ludwig, Leticia de Oliveira

**Affiliations:** 1 Molecular Biology and Biotechnology Department, Biosciences Institute, Federal University of Rio Grande do Sul, Porto Alegre, Brazil; 2 Institute of Humanities and Health, Fluminense Federal University, Niterói, Brazil; 3 Interdisciplinary Department, Federal University of Rio Grande do Sul, Tramandai, Brazil; 4 Management School, Federal University of Rio Grande do Sul, Porto Alegre, Brazil; 5 Department of Ecology, State University of Rio de Janeiro, Rio de Janeiro, Brazil; 6 Federal University of Pampa/UNIPAMPA, Uruguaiana, Brazil; 7 Department of Political Science, University of São Paulo, São Paulo, Brazil; 8 Physics Department, Federal University of Juiz de Fora, Juiz de Fora, Brazil; 9 Biomedical Institute, Fluminense Federal University, Niterói, Brazil

**Keywords:** Science, technology and society, Education

## Abstract

Worldwide, parenthood remains a major driver for the reduced participation of women in the job market, where discrimination stems from people’s biases against mothers, based on stereotypes and misconceptions surrounding the vision of motherhood in our society. In academia, parenthood may be perceived as negatively affecting scientists’ commitment and dedication, especially women’s. We conducted a survey amongst Brazilian scientists and found that mothers self-reported a higher prevalence of negative bias in their workplace when compared to fathers. The perception of a negative bias was influenced by gender and career status, but not by race, scientific field or number of children. Regarding intersections, mothers with less than 15 years of hiring reported having suffered a higher rate of negative bias against themselves. We discuss implications of these results and suggest how this negative bias should be addressed in order to promote an equitable environment that does not harm women in science.

## Introduction

Gender bias is predominant in science, especially in the STEM fields, where women’s presence decreases sharply during the academic career (Isphording, Qendrai, 2019). This phenomenon is known as the “scissors effect” (Areas et al., 2020) or “leaky pipeline” (Flaherty, 2018; Pell, 1996) and many studies showed that women mostly leave academia after graduate school at the post-doc level (Areas et al., 2020; Hill et al., 2010). The factors leading women to abandon academia are multiple, including a gendered workplace (Prieto-Rodriguez et al., 2022), lower funding (Van der Lee, Ellemers, 2015; Zandonà 2022), different forms of harassment (Clancy et al., 2014), implicit bias (Dutt et al., 2016; Eaton et al., 2020; Moss-Racusin et al., 2012), and, probably the most important one, motherhood (Machado et al., 2019; Morgan et al., 2021). Explicit and implicit bias against women in science can also be strong drivers causing women to leave academia for feeling or being considered unwelcoming, unfitting or not competent enough (Moss-Racusin et al., 2012). Here, we define bias as a construct that violates the principles of impartiality by implicating in prejudicial analysis or judgments that do not allow fair assessments (Staats et al., 2014). Often, this unfair judgment results in a negative impact on the evaluation of individuals or groups that are associated with social stereotypes of persons stigmatized as intellectually impaired or incapable (for a review, see Calaza et al., 2021).

Research shows that both women and men are biased against women in the academic environment (Roper, 2019). Bias against women are also evident in metrics used to evaluate academics, from impact factors to reference letters (Fortin et al., 2021), which in turn hurts women’s recognition and prestige (Oliveira et al., 2021). Most of the time, this bias can be implicit, that is, not explicitly perceived. One of the most rigorous studies on this subject has shown that, when assessing equivalent applications for a laboratory manager position, both men and women evaluators were more willing to mentor male candidates and ranked them as more competent. This biased assessment resulted in higher salary offers in relation to offers extended to female candidates (Moss-Racusin et al., 2012). Eaton et al. (2020) replicated the effects of gender bias in CV assessment, including its association with race/ethnicity. The authors found that Black women and Latin American candidates were ranked the lowest in the hiring process. This result suggested a strong combined effect of gender and race/ethnicity bias. Bias has cumulative effects on the scientific careers of women, including promotions and tenure, publications, grant application success, and respect (Roper, 2019).

The cognitive and motivational mechanisms behind gender bias are complex, but many studies document that motherhood triggers negative assumptions of competence and commitment towards women in the labor market, implying the well-known maternity penalty in women’s career (Fuegen et al., 2004; Heilman and Okimoto, 2008; Mavriplis et al., 2010; Okimoto and Heilman, 2012). This contrasts with no penalties or even an improvement in work-related evaluations that are received by fathers (Aranda and Glick, 2014; Correll et al., 2007; Luhr, 2020). A very interesting study simulated the application for a job and compared the assessment of equally qualified candidates—matching gender and race, so the groups being compared differed only in parental status. The experiment revealed that mothers were penalized in the process, being offered, for instance, a lower starting salary recommendation than women without children (Correll et al., 2007). Furthermore, competent and hard-working mothers tend to be considered less affectionate, less pleasant, and more interpersonally hostile, a phenomenon known as normative discrimination (Benard and Correll, 2010).

In the academic environment, STEM faculty members with young children are more likely to report the presence of ‘flexibility stigma’ (a term used to describe workplaces that punish those who do not fit the “ideal worker” profile). Those who report this stigma are more likely to decide towards leaving academia (Cech, Blair-Loy, 2014). The study of Mavriplis et al. (2010) showed the existence in academia of a negative bias towards those who seek to raise a family. However, studies about bias against parents are still scarce, especially considering self-perception of such bias. Subjective feelings of being discriminated against can have a great impact on the well-being of scientists, especially women, hindering the feeling of belonging and pushing women away from science. While it may seem obvious that parents face bias in academia, it is crucial to perform an analysis of this issue to establish a strong scientific foundation for understanding the phenomenon. While academia shares many features with other working environments, it has its own particularities. For example, a high proportion of academics and academic leaders reported frequently working more than 48 h a week, and the effect this had on satisfaction with work-life balance, when compared to professional services staff (Ryan and Peters, 2015). This highlights how academias’ distinct culture, values, and demands can create unique challenges for employees, particularly those with caregiving responsibilities. Therefore, the occurrence and perception of negative bias towards parents in academia in particular needs to be further investigated, as there could be multiple factors exacerbating the bias. The objective of this study was to evaluate the self-reported negative bias that scientists with children suffer in their work environment and to provide insights into the factors that influence this perception. For this, we developed an online survey to assess the perception of bias by faculties with children in their workplace, analyzing different variables in order to understand the possible intersections that could be contributing to exacerbate or decrease the perception of a negative bias. Studies have been expliciting an array of barriers and penalties mothers have been facing in the academic environment (Williams, 2005). Therefore, we hypothesize that female faculty with children perceive a greater negative bias than their male peers. We also hypothesize that factors such as race, career stage, and research area aggravate the self-perception of a negative bias, with Black, early career, and STEM faculty being the most affected. By providing quantitative data on a topic that has largely been explored using qualitative methods, we hope to have a more accurate and reliable understanding of the prevalence of bias against parents in academia.

## Methods

### Survey instrument

This study has been approved by the Ethics Committee. The questionnaire was developed to assess the perception of bias by researchers with children in their workplace. It consisted of 22 questions collecting information about the researchers’ demographics and seven questions using a 5-point Likert scale, where respondents were asked to rate their perceived negative bias, where 1 meant “I completely disagree”, and 5 “I completely agree”.

The full survey is provided in the Supplementary Material but, briefly, it is designed to investigate the perception of biases against parents in academia. The questionnaire gathers information on the demographic and academic profile of the respondent, including their gender, age, race, and education level. It also asks about their current position, years of experience, and involvement in graduate programs. The survey explores the respondent’s parenthood experience, such as the number of children, their age, and whether they parent children with disabilities. The questionnaire also contains statements related to the respondent’s experience of becoming a parent while working as faculty, covering topics such as the reception of leave request, changes in treatment after returning to work, pressure to take on additional tasks, perceptions of commitment and competence, job performance reviews, access to professional opportunities, and the need to constantly prove competence. Overall, the survey is testing the hypothesis that parents in academia experience biases that affect their work opportunities and performance towards career progression as well as which factors influence this perception.

### Sample

The survey was conducted via an online form, which was both promoted on social media and emailed to universities and research centers based in Brazil. The snowball sampling technique was also used, where existing study subjects recruited future subjects from among their acquaintances. The survey took approximately 5 minutes to complete and it was in Portuguese. Survey was opened between October 8th and November 27th 2021, and was answered by 995 Brazilian scientists across the country, resulting in a sample with varied demographic backgrounds; 105 respondents were excluded for not having had at least one child after being hired as faculty. The final sample was 890 participants.

### Analysis plan and statistics

Data collected through the survey was analyzed to investigate the perception of biases against parents in academia. We performed both descriptive and inferential statistical analyses to answer our research questions.

Descriptive analyses were used to characterize the sample regarding demographic and academic variables, as well as regarding the parenthood experience of the participants as faculty. Frequencies, percentages, means, and standard deviations were calculated, as appropriate. The initial analysis to compare mothers and fathers was performed using the scores for each question by respondent’s gender. Due to the nature of each likert question (i.e., ordinal variable), the comparisons were done using the Mann–Whitney test.

We also explored the reliability of the questionnaire as a single construct (self-perception of negative bias against parents). In this case, the score for each individual was used as the dependent variable (ranging from 5 to 35 points). We conducted bivariate tests such as Mann–Whitney or Kruskal–Wallis to pick which variables should be included in the Linear regression model as predictors. Some of the questions (i.e., number 1, 2, 4, 5, and 6) were re-coded as reverse scores and the total sum of the 7 items were then used in further analysis. Utilizing the whole sample (*n* = 890), we attained good internal reliability with a standardized cronbach’s alpha of 0.81.

Due to the low number of respondents in some categories of the *race* factor (1 indigenous; 13 asians; 15 that did not declare) our sample for the following statistical analyses consisted of 861 participants. That being said, for the linear regression model with the total score of the parental bias questionnaire (ranging from 5 to 35 points) as dependent variable, we conducted a few bivariate tests (i.e., Mann–Whitney or Kruskal–Wallis) to pick which variables should be included in the model as predictors. The variables tested were: gender (men or women), race (black or white), graduate advisor (yes or no), Productivity Scholarship (PS) holder (yes or no), number of children (one or more than one), research field, and hiring time (less or more than 15 years). Those variables with *p*-value less than or equal to 0.20 entered in the linear regression model. All the assumptions of the linear model were checked and met (e.g., normality of residuals, linearity and non-constant variance of error terms, absence of multicollinearity).

Inferential analyses were conducted to test the hypotheses of the study. We used linear regression models to assess the association between the perception of biases against parents and several demographic and academic variables. First, we built a model with only the main effects of gender, graduate supervisor and hiring time, with no interactions. Second, we built two other models with interaction terms, these are models 2 and 3. Model 2 consisted of the main effects plus the interaction term between gender and graduate supervisor; while model 3 consisted of the main effects plus the interaction term between gender and hiring time.

The analyses were conducted in RStudio (R Core Team, 2022) and a *p*-value of 0.05 or less was considered statistically significant. Packages *Tidyverse* (Wickham et al., 2019), *psych* (Revelle, 2022), *car* (Fox and Weisberg, 2019), and *moments* (Komsta and Novomestky, 2022) were used. The analysis script can be found in the supplementary material.

## Results

A detailed description of the survey respondents is provided in Supplementary Table 1. The total sample size was 890 faculty members that are parents, predominantly women (69.3%). In Brazil, women are approximately 50% among researchers, according to the last Brazilian National Council for Scientific and Technological Development (CNPq) Census (http://lattes.cnpq.br/web/dgp/censo-atual/). A prevalence of female respondents in studies targeting university faculty members have been previously reported (Smith, 2008).

Results are presented in separated sections, according to the analyses performed: group comparisons, bivariable analysis and regression analysis.

### The perception of bias amongst faculty with children

#### Group comparisons

**Fig. 1:**
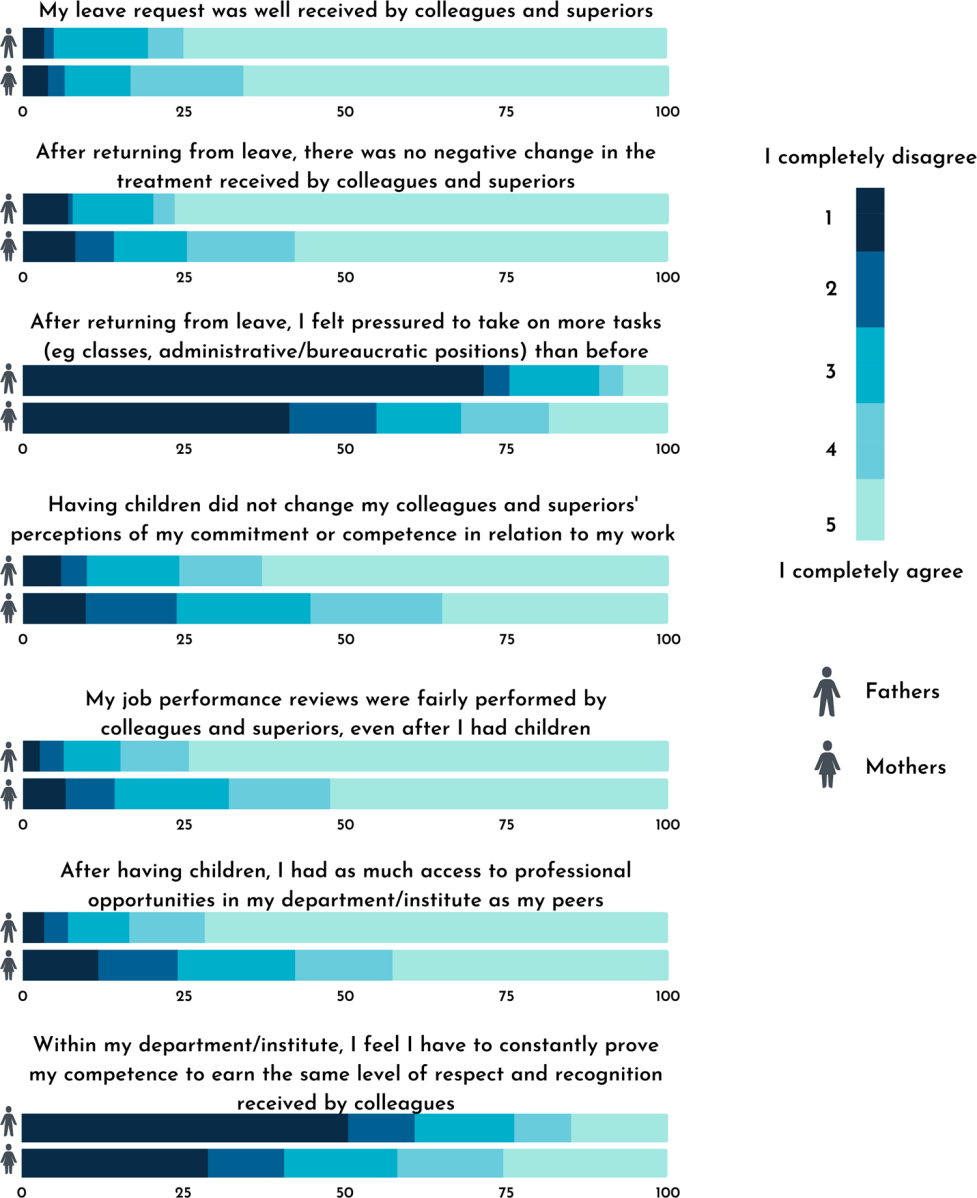
Self-perception of negative bias towards parents in academia. Results are shown in percentage by the reported gender of the respondents (male, top line; female, bottom line), using a 5-point Likert scale, where 1 meant “I completely disagree”, and 5 “I completely agree”.

First, we explore differences in bias perception among female and male faculty with children, as presented in Fig. 1, by summarizing the responses in percentages, segregated by gender. For all questions investigated in this study, we found statistical differences between mothers and fathers (Table 1).

**Table 1:** Statistical analysis of responses for each individual question, by gender.

	Mothers x Fathers (Mann–Whitney test)
My leave request was well received by colleagues and superiors.	*p* = 0.035
After returning from leave, there was no negative change in the treatment received by colleagues and superiors.	*p* < 0.001
After returning from leave, I felt pressured to take on more tasks (e.g., classes, administrative and/or bureaucratic positions) than before.	*p* < 0.001
Having children did not change my colleagues and superiors’ perceptions of my commitment or competence in relation to my work.	*p* < 0.001
My job performance reviews were fairly performed by colleagues and superiors, even after I had children.	*p* < 0.001
After having children, I had as much access to professional opportunities in my department/institute as my peers.	*p* < 0.001
Within my department/institute, I feel I have to constantly prove my competence to earn the same level of respect and recognition received by colleagues.	*p* < 0.001

Women were more likely to perceive bias as a result of maternity leave, reinforcing the occurrence of the flexibility stigma in academia. They also reported feeling more pressured to assume more tasks after returning from the leave, as opposed to their previous workload.

Mothers were more likely to report negative bias triggered by parenthood than fathers: 63% of fathers, but only 35% of mothers, completely agreed that having children did not change their colleagues’ and superiors’ perceptions of their commitment or competence at work. Also, fathers more frequently completely agreed (74.4% vs 52.4% of the mothers) that their performance was fairly evaluated by their colleagues/superiors.

Male scientists with children completely agreed that they had as much access to professional opportunities as their peers at a higher rate (71.8%) than mothers (42.8%). Also, 50.5% of fathers completely disagreed that they felt they had to constantly prove their competence in order to earn the same level of respect and recognition received by colleagues, while only 28.8% of mothers disagreed completely with the same statement.

#### Bivariable analyses

In addition to analyzing each question individually, we analyzed all questions together to obtain the perception of bias against parents in academia as a whole. Thus, we also explored the reliability of the questionnaire as a single construct, a self-perception of negative bias against parents.

When comparing the total score, regarding gender, we found a significant difference between men and women (*W* = 49540, *p* < 0.001), with women (mean = 15.87, SD = 6.6) scoring higher than men (mean = 11.96, SD = 4.83). This corroborates with our findings from the initial analysis with each separate question, showing a greater self-perception of bias against parents by women.

When comparing the total score between Black and White parents, no difference was found (*W* = 51033, *p* = 0.091), although we can see a trend of Black participants scoring a little higher (mean = 15.41, SD = 6.4) than White participants (mean = 14.56, SD = 6.37). The low presence of Black academics and, hence, respondents, could have impacted these results due to the low number of cases for analysis.

We also analyzed the perception of bias considering the career stage and status, here represented by time of hiring, and being a graduate supervisor and/or a Productivity Scholarship (PS) holder. These scholarships from the Brazilian National Council for Scientific and Technological Development are a reflection of prestige in the Brazilian higher education system. Having less than 15 years of hiring time was related to greater scores (mean = 15.26, SD = 6.39) when compared to those with 15 years or more (mean = 13.52, SD = 6.21) (*W* = 65900, *p* < 0.001). Participants who are graduate supervisors seem to have a lower total score (mean = 14.23, SD = 6.2) when compared to those who are not supervisors (mean = 16.45, SD = 6.73) (*W* = 75985, *p* < 0.001). In relation to those that are PS holders and those who are not, we found a significant difference (*W* = 72490, *p* < 0.001) showing that PS holders have lower scores (mean = 12.87, SD = 6.13) than those parents that do not possess such scholarship (mean = 15.17, SD = 6.37). Together, these results suggest that parents who have less than 15 years of employment time and who are not supervisors or PS holders are more likely to report negative parental bias.

When comparing research fields, we did not find any significant difference between groups (Chi-squared = 7.05, *p* = 0.423, df = 7). Parents working in Agricultural sciences (mean = 15.19, SD = 7.15), Biological sciences (mean = 14.06, SD = 6.2), Health sciences (mean = 14.78, SD = 6.63), Exact and earth sciences (mean = 14.73, SD = 6.33), Humanities (mean = 15.18, SD = 6.42), Social sciences (mean = 15.41, SD = 6.41), Engineering (mean = 13.29, SD = 5.68), and Linguistics, language and arts (mean = 14.85, SD = 5.64) had similar scores, indicating that the bias against parents may be a universal phenomenon in academia, even in fields with higher prevalence of female scientists.

Finally, there were no differences between parents with only one child (mean = 14.92, SD = 6.23) and those with more than one (mean = 14.56, SD = 6.5) (*W* = 95531, *p* = 0.234).

#### Regression analyses

**Fig. 2:**
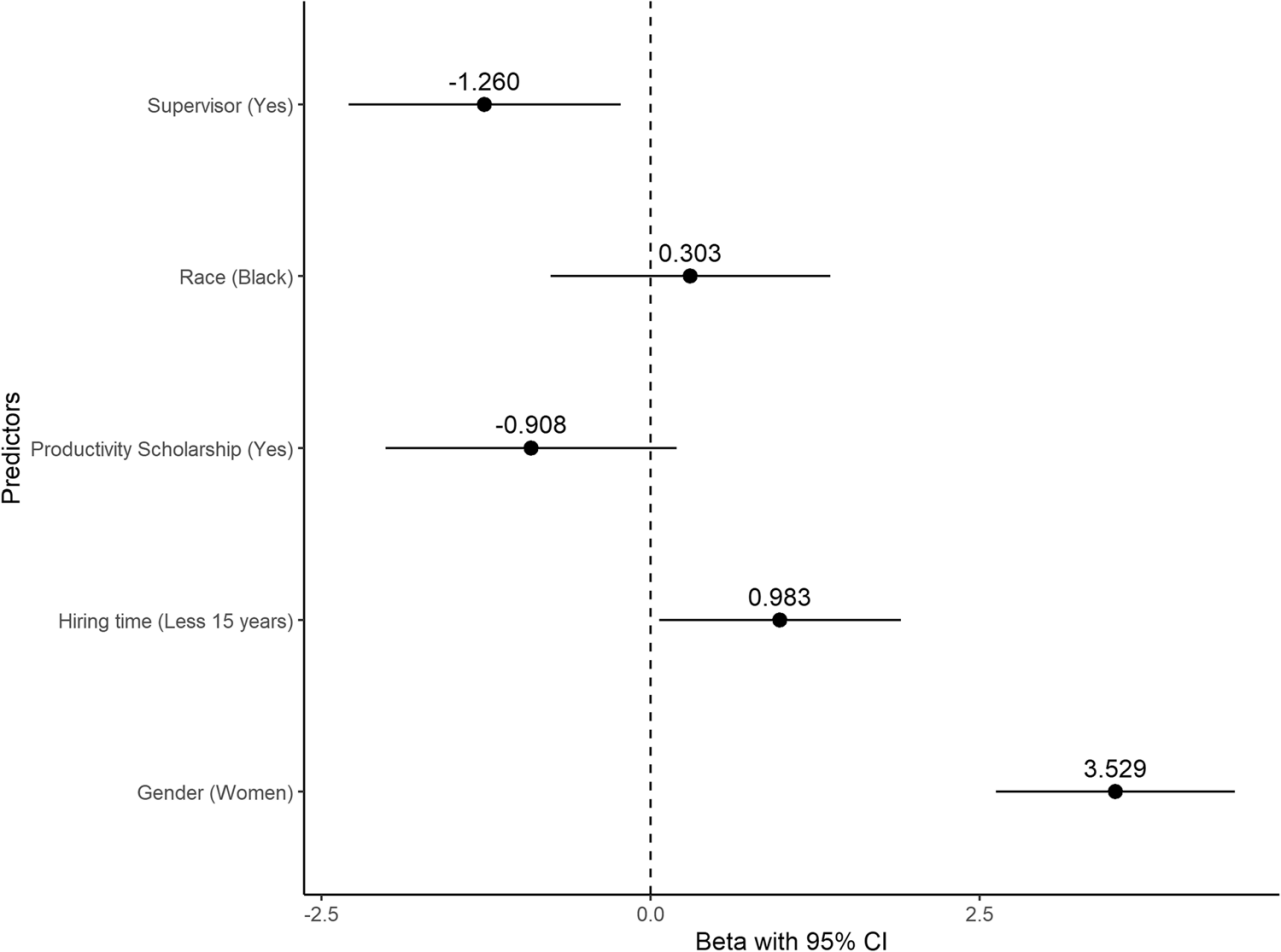
Total score predictors. Forest plot depicting the predictor’s estimates (95% confidence interval) of the regression model with the total score of self-perception bias against parents as the dependent variable.

When looking at the potential variables that could enter our linear model, we found that gender, race, graduate supervisor, PS holders and hiring time were related to the outcome. Fitting the model with these variables as predictors and the total score of self-perception bias against parents as the response variable, we found that the overall regression was statistically significant (adjusted *R*² = 0.09, *F* (5, 855) = 19.08, *p* < 0.001). We observed that gender was a significant predictor of the total score (*β* = 3.53, *p* < 0.001), suggesting that women score higher than men even with other possible confounding variables. Being a graduate supervisor was also a significant predictor (*β* = −1.26, *p* = 0.017), with those that responded yes scoring less than those who were not supervisors. Hiring time was a significant predictor also (*β* = 0.983, *p* = 0.036), with those parents with less than 15 years of time of hiring scoring higher than those with 15 years or more. Race (*β* = 0.303, *p* = 0.576) and PS holder (*β* = −0.908, *p* = 0.107) were not significant predictors of the total score of self-perception bias against parents (Fig. 2).

The model suggests that even with other possible predictors, only the variables gender, graduate supervisor, and hiring time played a considerable role in predicting the total score of the questionnaires, with gender having the greatest effect, as being a woman is associated with an increase of 3.53 points in the total score when compared to men (Table 2).

**Table 2:** linear regression model with the total score of the questionnaires as dependent variable.

	*β*	SE	95% CI		*t*-value	*p*-value
			Lower	Upper		
*Gender*						
Men	Reference					
Women	3.529	0.4627	2.621	4.437	7.627	0.000
*Race*						
White	Reference					
Black	0.303	0.5417	−0.760	1.366	0.559	0.576
*PS holder*						
No	Reference					
Yes	−0.908	0.5623	−2.011	0.196	−1.614	0.107
*Graduate supervisor*						
No	Reference					
Yes	−1.260	0.5260	−2.293	−0.228	−2.396	0.017
*Hiring time*						
15 years or more	Reference					
Less than 15 years	0.983	0.4683	0.064	1.902	2.099	0.036

Adjusted *R*-squared = 0.9511, *F*-statistics (5, 855) = 19.08, *p*-value < 0.001. *SE* standard error, *95% CI* confidence interval, *PS* productivity scholarship.

#### Intersecting factors

Since our main goal was to explore the intersection between gender and other possible predictors in the perception of negative bias against parents, we performed a few model comparisons including interaction terms between gender and the significant predictors of the main regression model (i.e, graduate supervisor and hiring time). The comparisons can be seen in Supplementary Table 2. We found that the model with the inclusion of the interaction term of gender and hiring time reduced the residual sum of squares and was statistically significant (*F* = 6.121, *p* < 0.05). Thus, for better evaluation of this interaction between the levels of gender and hiring time, we performed a series of pairwise comparisons (*t*-tests with corrected *p*-value using the *bonferroni* method).

Being a woman is associated with higher scores in the scale, and more specifically, women with less than 15 years of hiring time score 4.46 points higher than men with less than 15 years of hiring time (Table 3). Interestingly, women with 15 years or more of hiring time still score higher than men with less than 15 years of hiring time (mean difference = 2.5). Moreover, for fathers, the hiring time was not statistically significant, suggesting a specific effect on mothers.

**Table 3:** Pairwise comparisons of the interaction between gender and hiring time.

	Mean difference	SE	*p*-value*
Men 15 years or more – Women 15 years or more	−2.095	0.771	0.040
Men 15 years or more – Men Less than 15 years	0.406	0.784	1.000
Men 15 years or more – Women Less than 15 years	−4.057	0.691	<0.001
Women 15 years or more – Men Less than 15 years	2.501	0.667	0.001
Women 15 years or more – Women Less than 15 years	−1.962	0.554	0.002
Men Less than 15 years – Women Less than 15 years	−4.463	0.568	<0.001

*SE* standard error. *p-value corrected by *bonferroni* method.

## Discussion

We have shed light on the challenges that parents, especially mothers, face in academia due to the bias against them, which has been an under-explored area in research. Our results revealed a strong perception of negative bias against scientists with children, where mothers were more likely to report negative bias triggered by parenthood than fathers. Women were more likely to perceive bias as an effect of maternity leave and reported feeling more pressured to assume more tasks after returning from the leave and feeling they had to constantly prove their competence. On the other end of the spectrum, fathers more frequently completely agreed that their performance evaluations were fairly performed by their colleagues/superiors and that they had as much access to professional opportunities as their peers at a higher rate than mothers. Importantly, considering the questionnaire as a single construct, gender had the highest predictive value for the total score of the questionnaires. This gendered negative bias can be considered a micro-aggression, thus having negative consequences on women’s performance and permanence in academia, affecting not only mothers’ self-confidence, but also a very valuable asset: their objective assessment.

It is important to address the fact that, in our survey, we measured perception of the bias, not the bias itself. Nevertheless, since maternal bias has been proven to exist in other sectors (Arena et al., 2022; Correll et al., 2007), it may very well be that mothers are reflecting an accurate understanding of their work environment in academia. Academic workplaces are often perceived as being more progressive and open-minded than other workplaces, with a greater emphasis on intellectual and social diversity. However, this perception can mask underlying biases and stereotypes that can be especially harmful to certain groups, such as women with caregiving responsibilities, where their commitment and dedication, as well as their competence, are routinely questioned by colleagues and/or superiors. It reverberates in women finding themselves in a constant state of alert, burdened with heavier workload than their peers, having to prove themselves capable and productive at all times.

In Brazil, paid maternity leave varies from 120 to 180 days, while paternity leave ranges from 5 to 20 days. Asymmetric parental leave policies are common worldwide and perpetuate gender inequality, having significant, long-lasting outcomes for families, organizations, and the economy (Duffy et al., 2020). Our findings suggest that it may also contribute to the negative bias observed against mothers in science. Countries that provide long maternity and paternity leaves or that encourage mothers and fathers to share paid parental leave may build up to a different motherhood penalty scenario. According to this expectation, female CVs of candidates for an Associate Professor position in Northern European countries were perceived as more competent and hireable than equally qualified male candidates, and no evidence of motherhood penalty was observed (Carlsson et al., 2021).

We were very interested in investigating which factors, besides gender, could influence the perception of bias against parents. Race has been demonstrated to have an important intersection with gender when analyzing the participation of women in science and the impacts of the pandemic on women’s career in academia (Staniscuaski et al., 2021). In Brazil, only 23.6% of researchers are Black, despite the fact that Black people represent 54% of the Brazilian population. Black women account for only 3% of PhD supervisors (da Silva, 2010 (https://www.frontiersin.org/articles/10.3389/fpsyg.2021.663252/full#B20); Morcelle et al., 2019 (https://www.frontiersin.org/articles/10.3389/fpsyg.2021.663252/full#B55)). Surprisingly, race was not an influencing factor in the analysis performed in the present work. In a survey conducted by Williams et al. (2016), mothers from all race groups tended to attribute maternal wall problems to gender. Past studies showed that the motherhood penalty at work occurs regardless of race. A systematic review of Arena et al. (2022) found that motherhood wage penalty persists even when controlling for potentially confounding variables, such as race and number of children. Correll et al. (2007) demonstrated that, although African-American mothers were rated as less likely to be promoted than White mothers, both experienced a motherhood penalty of the same magnitude. Nonetheless, further in-depth research would be extremely important to deepen our understanding of the complex intersections between parenthood, gender, and race.

Previous work has shown that mothers in STEM disciplines perceive that they have to work harder than STEM and non-STEM fathers and mothers not in STEM disciplines, possibly because of the academic STEM’s masculine work culture (Kmec, 2013). However, a recent study has shown that gender bias is still prevalent in gender-balanced and female-dominated industries, including higher education (Stephenson et al., 2022 (https://www.emerald.com/insight/search?q=Amber%20L.%20Stephenson)). Here, we did not observe an effect of the field of knowledge on the extent parents perceived bias, nor on how mothers felt in their workplace overall.

Career stage and status seem to be an important factor on the perception of bias, where graduate supervisors were less likely to perceive negative bias for being parents. In Brazil, academics have to achieve very strict requirements to get a place in a graduate program as a supervisor. Therefore, our results suggest that career consolidation decreases the perception of negative bias against parents. We expected similar results considering the PS holders. In fact, considering the bivariate analyses, PS holders have lower scores in the perceived negative parental bias than those parents that are not PS holders. However, we were not able to find any significant results in the complete linear regression model. One possibility is that a sample size limitation (lower number of PS holders), in this case, prevents us from observing significant results. The results observed when evaluating career status could be a reflection of the fact that graduate supervisors/PS holders have a team who can continue their research while they are on leave or adjusting to their return after leave. On the contrary, without that, academics who are not graduate supervisors/PS holders may face even higher barriers in their careers after they return from leave.

In the present study, parents who have been hired for a shorter time have greater perception of negative bias. These early-career parents may suffer more serious consequences than parents at higher steps of the academic ladder, who have paved a longer career. Especially considering mothers, children reduce women’s participation in the workforce, but this effect is stronger when women are younger (Kahn et al., 2014). This effect was largely, but not entirely, explained by differences in productivity measured by accumulated work experience (Budig and England, 2001). Given this, it is possible that parents, especially mothers, with shorter hiring time in academia perceived a strong bias from their peers regarding their commitment and performance. The results of the present study showed that mothers were more likely to report negative bias triggered by parenthood than fathers, and most mothers felt that they had to constantly prove their competence. Even though work experience accumulated over time can alleviate self-perceived negative bias against mothers, it is important to highlight that our results showed that even with established careers in academia, mothers still perceive more negative bias when compared to fathers that have shorter hiring time. Therefore, mothers are more vulnerable to negative bias which, in turn, can impact their performance in academia.

While our study found that mothers face higher bias in academia, it is important to acknowledge that we cannot definitively state that the bias is solely because they are mothers and not also because they are women. There may be complex and intersectional factors at play, including societal norms and gender stereotypes that affect both mothers and women in general. However, our study’s focus on academics with children allowed us to examine the unique experiences of mothers in academia and provided insight into the specific challenges they face in balancing their personal and professional lives. Additionally, our study’s findings align with a growing body of literature that has identified motherhood as a significant factor in workplace discrimination and bias against women. For example, Williams (2005) suggests that a professor’s noticeably pregnant body may activate a set of biases she calls the ‘maternal wall’. This ‘attribution bias’ occurs when maternity becomes a trigger for the assumption that professors who are mothers cannot fit into the ‘24/7 ideal worker’ model. Therefore, while we acknowledge that our study has limitations, including the lack of a comparison group of academics without children, we believe that our conclusion that motherhood is a key factor generating bias in academia is supported by our data and is consistent with broader trends in the literature.

In order to challenge the bias against parents, especially mothers, found to exist in academia, we need to raise awareness and promote a major cultural shift in how motherhood is perceived, especially concerning the reconciliation of mothering and work. Structural changes in institutions are fundamental to valuing motherhood in academia and helping to eliminate the implicit bias against parents, especially mothers. Significant structural and institutional progress takes time and a lot of work, but it can be the most effective way to tackle the problem. Addressing individual and systemic bias is no easy task, but is a promising step to create a stronger scientific community (Metcalf, 2018). Within academic institutions, an raising-awareness approach is needed, ranging from data generation, which allows us to get to know the community and truly understand the problem, to the promotion of effective campaigns and actions that could put in motion an urge towards individual change in attitude (for a Roadmap for gender equality, see Schreiweis et al., 2019). Such campaigns and actions should focus on an open and frank discussion on the institution’s scenario comprising all sorts of bias. It should also aim at showing that this is a systemic problem, present in all instances within the institution. Self-knowledge of the entire academic community, but especially of those occupying decision-making positions, about implicit bias, gender stereotypes, and the intersection of the issue with parenthood and race, is key to promote changes. A previous study has shown that committees that acknowledged their potential bias promoted equitable numbers of men and women in a real-world promotion competition for research director positions in France, while committees that tended to disagree that gender discrimination contributes to women’s underrepresentation in STEM promoted fewer women (Regner et al., 2019). Implicit bias training may also be an option to reduce bias. While we can find evidence that it works (Stone et al., 2020), this is still up for debate, especially considering how this training is done (Forscher et al., 2019). Therefore, this kind of training must be well-designed and consider the peculiarities of each work environment to be effective. We also recommend training opportunities on issues related to parenthood—a subject usually ignored within academia, aiming at the deconstruction of myths around motherhood and fatherhood dynamics within the academic and scientific environment.

Another problem that urgently needs to be addressed in academia is the culture of overworking. In addition to impacting the mental health of the entire academic community, this culture implies a work-life imbalance that directly affects scientists with children, especially mothers, who cannot meet the (unrealistic) expectation of availability 24 h a day and, therefore, are seen and treated as less committed to their work. This leads to high pressure being put on them, by others and/or by themselves, resulting in a vicious circle of overwork and exhaustion. Actions and policies should also consider the parents’ career stage, and should strive in supporting parents at the beginning of their careers, for example, by reducing the workload during this stage to allow for career consolidation. While our study specifically focuses on the challenges faced by parents, it is possible that individuals without children may also face challenges in seeking time off or work-life balance. However, we believe that the challenges faced by parents are unique and warrant special attention, given the added responsibilities of caring for children.

We can not ignore the huge impact the pandemic has imposed in the academic career of women around the world, especially for mothers and those with caring responsibilities (Staniscuaski et al., 2020; 2021), not only on terms of their productivity (i.e., publishing papers) but also on their ability to establish critical workplace relationships. Care-taking responsibilities have increased for women since COVID-19 and the bias associated with these challenges has also increased (Reese, 2022). Tackling the impacts of the pandemic must necessarily include actions to mitigate bias against mothers in the workplace.

Transparent and systematic data collection and analysis procedures, from government and funding agencies to faculties, is essential to support the cultural change actions (including training toolkits, hiring manuals, etc.). It is important though, that any measure or policy implemented has its progress monitored over time (Roper, 2019), to assess specific interventions, to evaluate effectiveness of measures, to safely collect feedback and to correct the course of actions as needed.

Although data is important to expose the need for change in attitude, it might not be enough to persuade individuals completely. Therefore, institutions must promote effective and open-minded communication, alongside honest and straightforward conversations about the subject in order to revert the negative bias against parents in science.

## Supplementary information

Supplemental material

## Data Availability

The datasets generated during and/or analyzed during the current study are available from the corresponding author on reasonable request.

## References

[CR1] Aranda B, Glick P (2014). Signaling devotion to work over family undermines the motherhood penalty. Group Process Intergr Relat.

[CR2] Areas R, Abreu ARP, Santana AE et al. (2020) Gender and the scissors graph of Brazilian science: from equality to invisibility. Available at 10.31219/osf.io/m6eb4. Acessed Aug 08 2022

[CR3] Arena DF, Volpone SD, Jones KP (2022) Overcoming) Maternity bias in the workplace: a systematic review. J Manage 49:52–84. 10.1177/01492063221086243

[CR4] Benard S, Correll S (2010). Normative discrimination and the motherhood penalty. Gender Soc.

[CR5] Budig MJ, England P (2001). The wage penalty for motherhood. Am Sociol Rev.

[CR6] Calaza KC, Erthal FCS, Pereira MP (2021). Facing racism and sexism in science by fighting against social implicit bias: a Latina and Black Woman’s perspective. Front Psychol.

[CR7] Carlsson M, Finseraas H, Midtbøen AH, Rafnsdóttir GL (2021). Gender bias in academic recruitment? Evidence from a survey experiment in the Nordic region. Eur Sociol Rev.

[CR8] Cech EA, Blair-Loy M (2014). Consequences of flexibility stigma among academic scientists and engineers. Work Occup.

[CR9] Clancy KBH, Nelson RG, Rutherford JN (2014). Survey of academic field experiences (SAFE): trainees report harassment and assault. PLoS One.

[CR10] Correll SJ, Benard S, Paik I (2007). Getting a job: is there a motherhood penalty. Am J Sociol.

[CR11] Duffy S, van Esch P, Yousef M (2020). Increasing parental leave uptake: a systems social marketing approach. Australas Mark J.

[CR12] Dutt K, Pfaff DL, Bernstein AF (2016). Gender differences in recommendation letters for postdoctoral fellowships in geoscience. Nat Geosci.

[CR13] Eaton AA, Saunders JF, Jacobson RK (2020). How gender and race stereotypes impact the advancement of scholars in STEM: professors’ biased evaluations of physics and biology post-doctoral candidates. Sex Roles.

[CR14] Flaherty K (2018) The Leaky Pipeline for Postdocs: A study of the time between receiving a PhD and securing a faculty job for male and female astronomers. https://arxiv.org/abs/1810.01511. Acessed 24 Apr 2023

[CR15] Forscher PS, Lai CK, Axt JR (2019). A meta-analysis of procedures to change implicit measures. J Pers Soc Psychol.

[CR16] Fortin J, Bartlett B, Kantar M (2021). Digital technology helps remove gender bias in academia.. Scientometrics.

[CR21] Fox J, Weisberg S (2019) An {R} Companion to applied regression, third edition. Thousand Oaks CA: Sage. URL: https://socialsciences.mcmaster.ca/jfox/Books/Companion

[CR17] Fuegen K, Biernatm M, Haines E (2004). Mothers and fathers in the workplace: how gender and parental status influence judgments of job-related competence. J Soc Issues.

[CR18] Heilman ME, Okimoto TG (2008). Motherhood: a potential source of bias in employment decisions. J Appl Psychol.

[CR19] Hill C, Corbett C, St Rose A (2010) Why so few? Women in science, technology, engineering, and mathematics. AAUW, Washington, DC

[CR20] Isphording I, Qendrai P (2019) Gender differences in student dropout in STEM. IZA Research Reports 87, Institute of Labor Economics (IZA). Available at https://ftp.iza.org/report_pdfs/iza_report_87.pdf. Acessed Aug 08 2022

[CR22] Kahn JR, García-Manglano J, Bianchi SM (2014). The motherhood penalty at midlife: long-term effects of children on women’s careers. J Marriage Fam.

[CR23] Kmec JA (2013). Why academic STEM mothers feel they have to work harder than others on the job. Int J Gend. Sci Technol.

[CR24] Komsta L, Novomestky F (2022). _moments: Moments, Cumulants, Skewness, Kurtosis and Related Tests_. R package version 0.14.1, https://CRAN.R-project.org/package=moments

[CR25] Luhr S (2020). Signaling parenthood: managing the motherhood penalty and fatherhood premium in the U.S. service sector. Gend Soc.

[CR26] Machado LS, Perlin M, Soletti RC et al. (2019) Parent in science: The impact of parenthood on the scientific career in Brazil. In 2019 IEEE/ACM 2nd International Workshop on Gender Equality in Software Engineering (GE), IEEE (pp. 37–40)

[CR27] Mavriplis C, Heller RS, Beil C (2010). Mind the gap: women in STEM career breaks. J Technol Manag Innov.

[CR28] Metcalf H (2018). Creating a stronger STEM community by addressing our bias. Nat Hum Behav.

[CR29] Morcelle V, Freitas G, Ludwig ZMDC (2019). From school to university: an overview on stem (science, technology, engineering and mathematics) gender in Brazil. Quarks Braz Electron J Phys Chem Mater Sci.

[CR30] Morgan AC, Way SF, Hoefer MJ (2021). The unequal impact of parenthood in academia.. Sci Adv.

[CR31] Moss-Racusin CA, Dovidio JF, Brescoll VL (2012). Science faculty’s subtle gender biases favor male students. Proc Natl Acad Sci USA.

[CR32] Okimoto TG, Heilman ME (2012). The “bad parent” assumption: how gender stereotypes affect reactions to working mothers. J Soc Issues.

[CR33] Oliveira LD, Reichert F, Zandonà E (2021). The 100,000 most influential scientists rank: the underrepresentation of Brazilian women in academia. An Acad Bras Ciênc.

[CR34] Pell AN (1996). Fixing the leaky pipeline: women scientists in academia. J Anim Sci.

[CR35] Prieto-Rodriguez E, Sincock K, Berretta R (2022). A study of factors affecting women’s lived experiences in STEM. Humanit Soc Sci Commun.

[CR36] R Core Team (2022) R: a language and environment for statistical computing. R Foundation for Statistical Computing, Vienna, Austria. URL https://www.R-project.org/

[CR37] Reese (2022) The COVID-19 gender gap: Addressing bias at work can help bring women back to the office. Available at https://www.techrepublic.com/article/covid-19-gender-gap-addressing-bias-work-can-bring-women-back-office/. Aug 08 2022

[CR38] Régner I, Thinus-Blanc C, Netter A (2019). Committees with implicit biases promote fewer women when they do not believe gender bias exists. Nat Hum Behav.

[CR39] Revelle W (2022) psych: Procedures for personality and psychological research, Northwestern University, Evanston, Illinois, USA. Available at https://CRAN.R-project.org/package=psych Version = 2.2.5. Accessed Aug 08 2022

[CR40] Roper RL (2019). Does gender bias still affect women in science?.. Microbiol Mol Biol Rev.

[CR41] Ryan MK, Peters K (2015) Leadership and work-life balance. The Leadership Foundation for Higher Education, London

[CR42] Schreiweis C, Volle E, Durr A (2019). A neuroscientific approach to increase gender equality. Nat Hum Behav.

[CR43] da Silva J (2010). Doutoras professoras negras: O que nos dizem os indicadores oficiais. Perspectiva.

[CR44] Smith WG (2008) Does gender influence online survey participation? A record-linkage analysis of university faculty online survey response behavior. Online submission

[CR45] Staats C, Capatosto K, Wright R et al. (2014) State of the science: implicit bias review. Columbus, OH: Kirwan Institute for the Study of Race and Ethnicity. Available at https://kirwaninstitute.osu.edu/sites/default/files/pdf/2015-implicit-bias-review.pdf. Aug 08 2022

[CR46] Staniscuaski F, Reichert F, Werneck FP (2020). Impact of COVID-19 on academic mothers. Science.

[CR47] Staniscuaski F, Reichert F, Zandonà E (2021). Time to fight the pandemic setbacks for caregiver academics. Nat Hum Behav.

[CR48] Staniscuaski F, Kmetzsch L, Soletti RC (2021). Gender, race and parenthood impact academic productivity during the COVID-19 pandemic: from survey to action. Front Psychol.

[CR49] Stephenson AL, Dzubinsk LM, Diehl AB (2022). A cross-industry comparison of how women leaders experience gender bias. Pers Rev.

[CR50] Stone J, Moskowitz GB, Zestcott CA (2020). Testing active learning workshops for reducing implicit stereotyping of Hispanics by majority and minority group medical students. Stigma Health.

[CR51] Van der Lee R, Ellemers N (2015). Gender contributes to personal research funding success in the Netherlands. Proc Natl Acad Sci USA.

[CR52] Wickham H, Averick M, Bryan J et al. (2019) Welcome to the tidyverse. J Open Source Softw 4(43):1686. 10.21105/joss.01686

[CR53] Williams JC, Phillips KW, Hall EV (2016). Tools for change: Boosting the retention of women in the STEM pipeline.. J Res Gend Stud.

[CR54] Williams JC (2005) Work and family perspectives from research university faculty. New Dir Higher Edu 2005(130):67–80

[CR55] Zandonà E (2022). Female ecologists are falling from the academic ladder: a call for action. Perspect Ecol Conserv.

