# Supplementary material for: Bias against parents in science hits women harder
Source: Humanit Soc Sci Commun. 2023 May 4;10(1):201. doi: 10.1057/s41599-023-01722-x (PMC10159222; doi:10.1057/s41599-023-01722-x)
Supplement: Supplementary file 1 — Supplemental material [file 41599_2023_1722_MOESM1_ESM.docx]

**Supplementary material**

**Bias against parents in science hits women harder**

Fernanda Staniscuaski^1,*^, Arthur V. Machado^2^, Rossana C. Soletti^3^, Fernanda Reichert^4^, Eugenia Zandonà^5^, Pamela B. Mello-Carpes^6^, Camila Infanger^7^, Zelia M.C Ludwig^8^ & Leticia de Oliveira^9^

^1^Molecular Biology and Biotechnology Department, Biosciences Institute, Federal University of Rio Grande do Sul, Brazil.

^2^Institute of Humanities and Health, Fluminense Federal University, Brazil

^3^Interdisciplinary Department, Federal University of Rio Grande do Sul, Brazil.

^4^Management School, Federal University of Rio Grande do Sul, Brazil.

^5^Department of Ecology, State University of Rio de Janeiro, Brazil.

^6^Federal University of Pampa/UNIPAMPA, Brazil.

^7^Department of Political Science, University of São Paulo, Brazil.

^8^Physics Department, Federal University of Juiz de Fora, Brazil.

^9^Biomedical Institute, Fluminense Federal University, Brazil.

* Corresponding author. Fernanda Staniscuaski

**Email:**  fernanda.staniscuaski@ufrgs.br

**The complete version of the questionnaire “Perception of biases against parents in academia”**

1. E-mail address
2. Do you agree to participate in the survey? Yes/No
3. Do you confirm the birth or adoption of at least one child after being hired as faculty in the Brazilian higher education system? Yes/No

**Demographic and academic profile**

1. Date of birth
2. How do you identify yourself?

☐ Male

☐ Female

☐ Non-binary

1. Are you a transgender person? Yes/No
2. According to the Brazilian Institute of Geography and Statistics (IBGE) census race/color categories, you declare yourself:

☐ Indigenous

☐ Asian

☐ Black

☐ Parda

☐ White

☐ Prefer not to declare

1. In which region do you live?

☐ North

☐ Northeast

☐ Centerwest

☐ Southeast

☐ South

1. What is your highest education degree?

☐ Bachelor

☐ Master

☐ PhD

1. In which year was the highest degree obtained? ___________
2. Current position

☐ Professor at a public institution

☐ Professor at a private institution

☐ Professor at a community institution

☐ Other

1. Year of hiring as a professor at the current institution. _________
2. Are you associated with a Graduate Program (acting as a graduate supervisor)?

☐ Yes, as a collaborator

☐ Yes, as a professor

☐ Yes, as a visiting professor

☐ No

1. Are you a CNPq productivity scholarship (PS) holder?

☐ No

☐ Level 2

☐ Level 1D

☐ Level 1C

☐ Level 1B

☐ Level 1A

☐ Senior level

1. What is your area of knowledge?

☐ Agricultural Sciences

☐ Biological Sciences

☐ Health Sciences

☐ Humanities

☐ Social Sciences

☐ Linguistics, Language and Arts

☐ Exact and Earth Sciences

☐ Engineering

**Parenthood**

1. Number of children. _________
2. Are you a parent of a person with a disability? Yes/No
3. If you are a parent of a person with a disability, have you requested a workload reduction due to this condition? Yes/No
4. Youngest child's birth year. _____
5. If applicable, enter your other children's birth year. ______________
6. Did you take maternity leave, paternity leave or adopter leave? In the case of more than one child, answer considering your youngest child.
7. What period of leave have you been granted? In the case of more than one child, answer considering your youngest child. Only consider the period of formal leave as stated in your institutional records, not including vacations or days off.

**Your parenthood experience of as a faculty**

In this section, answer based on your experience of becoming a mother or father while working as faculty in a Brazilian higher education institution, stating how much you agree with the sentences below. Please respond on a scale of 1 to 5, with 1 being completely disagree and 5 being completely agree. Note: The term "leave" in the sentences below refers to maternity leave, paternity leave, and adopter leave.

1. My leave request was well received by colleagues and superiors
2. After returning from leave, there was no negative change in the treatment received by colleagues and superiors
3. After returning from leave, I felt pressured to take on more tasks (eg classes, administrative/bureaucratic positions) than before
4. Having children did not change my colleagues and superiors' perceptions of my commitment or competence in relation to my work
5. My job performance reviews were fairly performed by colleagues and superiors, even after I had children
6. After having children, I had as much access to professional opportunities in my department/institute as my peers
7. Within my department/institute, I feel I have to constantly prove my competence to earn the same level of respect and recognition received by colleagues
8. If you wish, please leave any comment and/or suggestion here.

**Supplementary table 1: Characterization of the study sample**

|  | General (%, n) | Male (%, n) | Female (%, n) |
| --- | --- | --- | --- |
| **Gender** |  | 30.7 (273) | 69.3 (617) |
|  |  |  |  |
| **Number of children**  One  Two  Three  Four  **Race/Ethnicity^§^** | 43.9 (391)  44.8 (399)  9.9 (88)  1.3 (12) | 39.6 (108)  45.4 (124)  11.7 (32)  3.3 (9) | 45.9 (283)  44.6 (275)  9.1 (56)  0.5 (3) |
| White  Black  Asian  Indigenous  ND* | 78.9 (702)  17.8 (159)  1.5 (13)  0.1 (1)  1.7 (15) | 77.6 (212)  15.4 (42)  1.8 (5)  0.4 (1)  4.8 (13) | 79.4 (490)  19.0 (117)  1.3 (8)  0 (0)  0.3 (2) |
| **Origin (Brazilian Region)**^+^ |  |  |  |
| North  Northeast  Center-west  Southeast  South | 2.8 (25)  14.7 (131)  4.6 (41)  45.4 (404)  32.5 (289) | 1.5 (4)  13.2 (36)  3.7 (10)  49.5 (135)  32.2 (88) | 3.4 (21)  15.4 (95)  5 (31)  13.6 (269)  32.6 (201) |
| **Scientific Area^£^** |  |  |  |
| Agricultural Sciences  Biological Sciences  Engineering  Exact and Earth Sciences  Health Sciences  Humanities  Linguistics, Language and Arts  Social Sciences | 7.2 (64)  17.1 (152)  7 (62)  17.5 (156)  19.8 (176)  13.5 (120)  4.4 (39)  13.6 (121) | 9.5 (26)  20.9 (57)  10.3 (28)  23.4 (64)  8.8 (24)  9.5 (26)  4.4 (12)  13.2 (36) | 6.2 (38)  15.4 (95)  5.5 (34)  14.9 (92)  24.6 (152)  15.2 (94)  4.4 (27)  13.8 (85) |

General data are shown as percentages (%) of the total number of respondents.

Gender data are shown as percentages (%) of respondents of the same gender (male or female).

The total number of respondents from each category is presented as (n).

^§^Terminology follows the official Brazilian census and the Brazilian Institute of Geography and Statistics (IBGE). Race/ethnicity categories are based on a skin color continuum ranging from very fair to very dark skin. We adopt official IBGE categories in the questionnaires: branca (White), preta (Black), parda, amarela (Yellow: translated as Asian) and indigena (Indigeneous). In Brazil, there is a common distinction between people who identify as Black (dark-skin Black people) and parda (light-skin Black people). In all results presented in the report, the Black category refers to both IBGE categories (preta and parda) together.

*Prefer not to disclose

^+^The percentage of researchers for each region in Brazil, according to the last Brazilian National Council for Scientific and Technological Development (CNPq) Census, is 6.3% (North), 20.5% (Northeast), 7.7% (Center-west), 42.5% (Southeast) and 22.9% (South).

^£^Scientific area nomenclature according to the CNPq classification.

**Supplementary table 2: model comparisons**

| **Models** | **RSS (df)** | **F** | **P value** |
| --- | --- | --- | --- |
| **Model 1 vs model 2** |  |  |  |
| Model 1 | 31643 (857) | 0.2215 | 0.638 |
| Model 2 | 31635 (856) |  |  |
|  |  |  |  |
| **Model 1 vs Model 3** |  |  |  |
| Model 1 | 31643 (857) |  |  |
| Model 3 | 31418 (856) | 6.121 | **0.0135** |

**Notes:** Model 1 consists in the total score as dependent variable and gender, graduate supervisor and hiring time as independent variables, with no interactions. Model 2 includes the same IV of the first model with the addition of the interaction term between gender and graduate supervisor. Model 3 includes the same IV of the first model with the inclusion of the interaction term between gender and hiring time. RSS = Residual Sum of Squares; df = degrees of freedom.
